# Comparison of the value of the STOP-BANG questionnaire with oxygen desaturation index in screening obstructive sleep apnea in Germany

**DOI:** 10.1007/s11325-022-02727-7

**Published:** 2022-10-21

**Authors:** Yan Wang, Ingo Fietze, Matthew Salanitro, Thomas Penzel

**Affiliations:** 1 Interdisciplinary Sleep Medicine Center, Charité–Universitätsmedizin Berlin, Luisenstrasse 13, 10117 Berlin, Germany; 2 The Federal State Autonomous Educational Institution of Higher Education, I.M Sechenov First Moscow State Medical University of the Ministry of Health of the Russian Federation, Moscow, Russia

**Keywords:** Obstructive sleep apnea, STOP-Bang questionnaire, Apnea–hypopnea index, Oxygen saturation index

## Abstract

**Purpose:**

Despite polysomnography being the gold standard method of diagnosing obstructive sleep apnea (OSA), it is time-consuming and has long waiting lists. Alternative methods including questionnaires and portable sleep devices have been developed to increase the speed of diagnosis. However, most questionnaires such as the STOP-BANG questionnaire (SBQ) are limited due to low specificity. This study evaluated the value of SBQ to screen for OSA and compared it with the oxygen desaturation index (ODI) and their combination.

**Methods:**

This retrospective study included patients who completed the SBQ and underwent a night at the sleep lab or home sleep testing. The ODI was extracted from these sleep study reports. The combination of SBQ with ODI and their individual scores were compared with apnea–hypopnea index (AHI) in terms of their accuracy in diagnosing OSA. Sensitivity, specificity, and area under the curve (AUC) for different severities of OSA were calculated and compared.

**Results:**

Among 132 patients, SBQ showed a sensitivity of 0.9 and a specificity of 0.3 to screen for OSA. As the severity of OSA increased, the sensitivity increased whilst specificity decreased for both measurements. ODI achieved an increased specificity of 0.8 and could correctly diagnose OSA 86% of the time which was better than SBQ’s 60%. For all severities of OSA, ODI alone displayed a larger AUC than SBQ and similar AUC to their combination.

**Conclusion:**

ODI produced a higher specificity and AUC than SBQ. Furthermore, ODI combined with SBQ failed to increase diagnostic value. Therefore, ODI may be the preferred way to initially screen patients for OSA as an easy-to-use alternative compared to SBQ.

## Introduction

Obstructive sleep apnea (OSA) is a sleep-related breathing disorder (SBD) characterized by intermittent obstruction and cessation of airflow in the upper airway (apnea). It can also be characterized by the temporary narrowing of the airway which results in the reduction of airflow (hypopnea). The narrowing is caused by the relaxation of throat muscles during sleep [1]. The prevalence of OSA in adults from the general population ranges from 9 to 38% [2] and it has been shown to be a risk factor for cardiovascular diseases [3], such as heart failure and hypertension. According to the Wisconsin cohort study [4], approximately 75% of people with SBD remain undiagnosed which suggests that OSA — being one of the main types of SBD — is currently highly undiagnosed in the general population.

The gold standard examination for diagnosing OSA is overnight polysomnography (PSG) in a sleep laboratory. PSG can accurately monitor sleep, blood oxygen levels, respiratory effort and airflow, limb movements, heart rate, and body position. However, the waiting lists can be quite long due to limited bed space [5]. The more common method of diagnosis is the Home Sleep Apnea Test (HSAT) which entails a more simplified 6-channel version of PSG [6]. Both PSG and HSAT can measure the frequency of apnea and hypopneas over the whole night to yield the apnea–hypopnea index (AHI)/respiratory event index (REI), and the oxygen desaturation index (ODI) [7]. The AHI determines whether an apnea or hypopnea has occurred from obstruction of airflow event in a sleep recording, and REI, which is used in HSAT, is a surrogate for AHI in PSG [8]. ODI was defined as the average number of desaturation episodes per hour of recording, with oxygen desaturation defined as a decrease in blood oxygen saturation (SpO2) by more than 3% below baseline. When breathing is obstructed during sleep, the blood oxygen levels drop. Therefore, it can identify apnea and hypopneas well [9].

There are some limitations to using PSG and HSAT. One is the first-night effects of sleeping in a laboratory connected to PSG or at home with HSAT. Both tests can increase sleep onset latency and reduce sleep efficiency which can affect the AHI score [10]. In this case, it is normal for patients to stay multiple nights in the lab to habituate to the new environment. This can be time-consuming and bay be costly, ausing PSG and HSAT to be relatively inaccessible to many patients [5, 11, 12].

Cheaper and less time-consuming methods of evaluating possible OSA are questionnaires such as the Berlin questionnaire, STOP-BANG (snoring, tiredness, observed apneas, blood pressure, body mass index, age, neck circumference, gender) questionnaire (SBQ), and the NoSAS (neck, obesity, snoring, age, sex) [13–15]. A systematic review by Abrishami et al. [16] showed that the SBQ was one of the best predictors of moderate to severe OSA by displaying the highest sensitivity and methodological validity compared to other questionnaires [16]. The SBQ is a relatively quick procedure that can inform a health professional of the severity of OSA. This is especially important due to the high frequency of undiagnosed people with OSA [5]. The main issue with the SBQ is that it lacks high specificity, especially for mild OSA [17, 18]. It is possible that SBQ could be improved by combining the SBQ with other diagnostic tools such as ODI. However, the value of SBQ and ODI needs further validation to show that the specificity can be improved.

Therefore, in order to enhance the speed and accuracy of OSA diagnosis in sleep medicine outpatient departments, the following aims were investigated: (a) testing the strength of the individual and the combined SBQ and ODI with AHI when diagnosing OSA, and (b) testing both the individual and the combined validity of the ODI and SBQ with the AHI when diagnosing OSA. It was expected that either ODI alone or in combination with SBQ would have higher specificity and greater area under the curve (AUC) than SBQ alone.

## Methods

### Participants and procedure

The ethics committee of the Charité–Universitätsmedizin Berlin approved the study protocol, and informed consent was obtained from all patients.

**Fig. 1:**
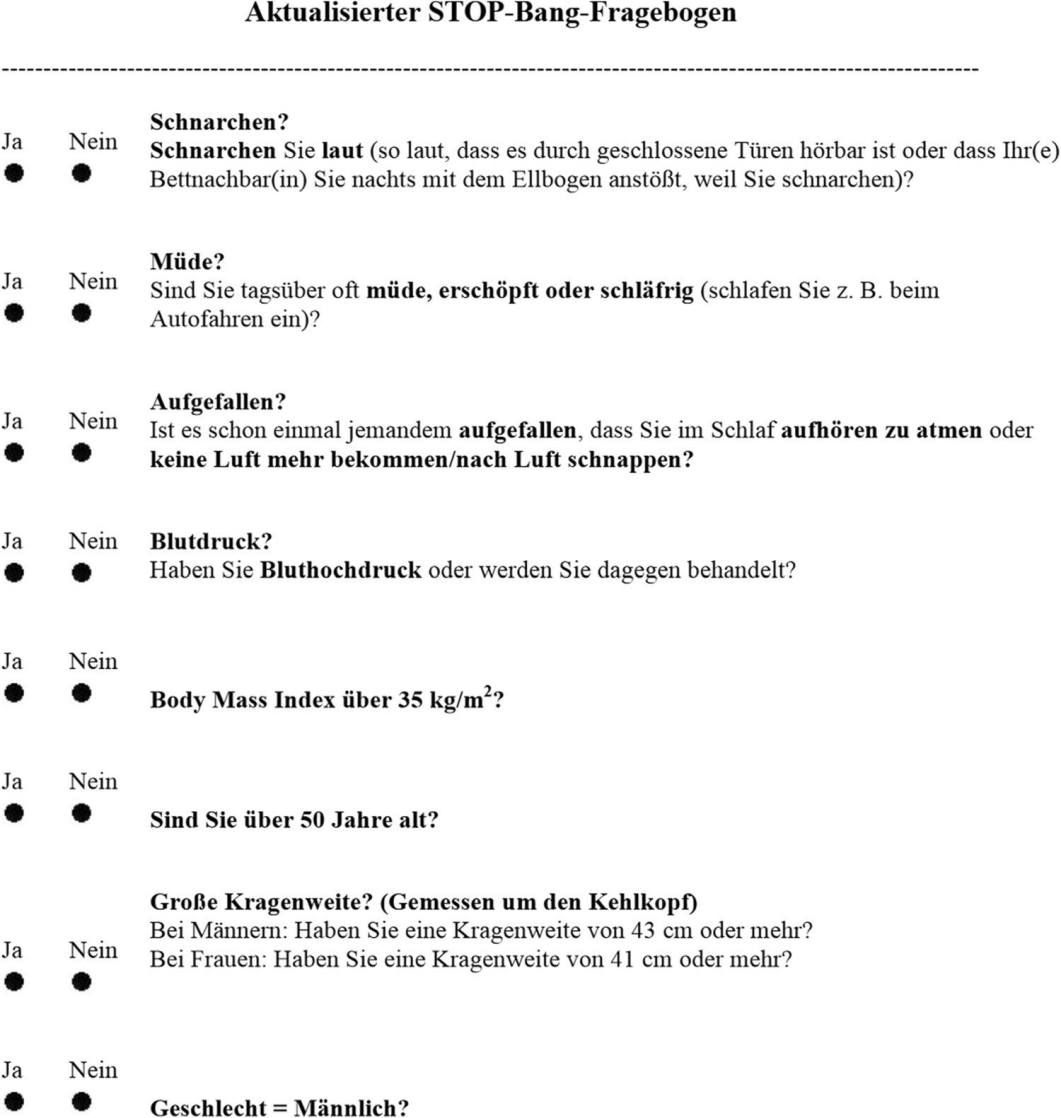
German version STOP-Bang questionnaire

During the first visit, all patients completed the previously validated German version of the SBQ [19, 20], and assistance was given by nurses if needed (see Fig. 1). Then each patient underwent either an FDA-approved in-lab PSG or HSAT. This recorded EEG, EOG, EMG, ECG, nasal pressure, rib cage, abdominal movements, body position, snoring sound, oximetry, and airflow. The filter settings that applied were in accordance with the American Academy of Sleep Medicine (AASM) manual recommendations. After these recordings were manually scored, the AHI/REI and ODI were extracted. Apneas were defined as an airflow drop of 90% during 10 s and hypopneas were defined as an airflow drop of 30% for 10 s associated with a blood desaturation of 3%. Both REI and AHI were then calculated (events/hour of recording time) to identify patients with either mild OSA was defined (5 ≤ AHI < 15 events/h), moderate OSA (15 ≤ AHI < 30 events/h), or severe OSA (AHI ≥ 30 events/h). All other data such as basic demographics (BMI, age, neck circumference, and sex) and sleep study data were collected retrospectively from the medical records. At the time of scoring the SBQ, the researchers were blinded from the identity of the patient to remove potential biases.

### Materials

The SBQ consists of 8 yes/no questions, each negative answer is scored as 1 and each positive is scored as 0. A total score of ≥ 3 but < 5 is classified as having an intermediate risk of OSA, and a high risk of OSA is between 5 and 8 points. High sensitivity can be seen when using a cut-off score of ≥ 3 with the AHI when defining OSA: 84% in detecting mild, 93% in detecting moderate to severe, and 100% in detecting severe. However, their specificities were low at 56%, 43%, and 37%, respectively [23]. As mentioned previously, the main parameters used to examine HSAT and PSG data were REI/AHI and ODI. The ODI was used to measure each patient’s desaturation episodes per hour, the severity of ODI was classified as either mild, moderate, or severe, and ODI < 5 was considered as having no problematic oxygen desaturation, and thus, we set the cut-off ODI value of 5 in this study.

### Data analysis

All analyses were computed in SPSS software version 22.0. A Spearman’s correlation coefficient was calculated to measure the strength of the relationship between the AHI with SBQ, ODI, and their combination. This was to identify whether each measure follows in the same direction as AHI.

An additional Spearman’s correlation coefficient was conducted to measure the strength of the relationship between different levels of AHI severity with SBQ, ODI, and their combination. This was to identify whether different severity levels of AHI are more or less related to each measure.

Positive predictive value (PPV) and negative predictive value (NPV) were calculated to validate the sensitivity and specificity of the cut-off values SBQ ≥ 3, ODI ≥ 5, and their combination. We also tested a model that combines ODI with the SBQ by logistic regression. This will inform us whether the combination increases the sensitivity and specificity of each measure.

In addition to this, receiver operating characteristic curves (ROCs) were performed to assess the diagnostic value of the ODI alone, SBQ alone, the combination of the two, and the logistic regression for different severities of AHI. The AUC produced from the ROCs range from 0.50 (no diagnostic ability) to 1.0 (perfect diagnostic ability) and can also be confirmed with a Youden statistic. The closer the value is to 1, the higher the diagnostic value the analysis holds. The differences in AUC calculated by the ROC characteristics were compared by *Z*-tests to assess their overall performance. *Z*-tests are a nonparametric test for 2 independent samples. They were used to compare the AUC characteristic derived from the same cases. Furthermore, the optimal cut-off values of ODI and SBQ in the AUC analysis were evaluated to achieve the most accurate prediction of OSA diagnosis (AHI ≥ 5, 15, and 30).

## Results

### Patient characteristics

The sample consisted of 224 patients who visited the outpatient department in the interdisciplinary sleep medicine center at the Charite-Universitatsmedizin in Berlin from January 2017 to January 2018. Ninety-two patients were excluded due to either incomplete follow-ups, SBQs, and/or clinical data, which left 132 patients to be included in this study. The demographic and mean SBQ responses from the patients are shown in Table 1. This retrospective study enrolled 132 patients, 74% were male and 61% were over 50 years old. Forty-nine patients were given HSAT and the other 83 patients were given PSG. The total sample had a mean AHI of 11.68 ± 15.02 event/h, a BMI of 28 ± 5.38 kg/m^2^, and an ODI of 13.4 ± 16.32. All patients reported at least one suspected OSA symptom, the most common were snoring (72%) and hypertension (47%). From this sample, 53% were diagnosed with OSA (mild 49%, moderate 30%, and severe 21%) and 47% with no OSA.

**Table 1:** Demographic data and STOP-Bang questionnaire characteristics

Characteristics	*n* = 132
Basic demographic data	
Sex (%); male	98 (74%)
Age, year	53.5 ± 14.0
BMI, kg/m^2^	28.0 ± 5.4
ODI/h	13.4 ± 16.3
AHI, event/h	11.7 ± 15.0
STOP-Bang questionnaires	
Age ≥ 50, year	81 (61%)
BMI ≥ 35, kg/m^2^	13 (10%)
Snoring	95 (72%)
Tiredness	76 (58%)
Observed apnea	62 (47%)
Hypertension	62 (47%)
Neck circumference ≥ 40 cm	40 (30%)
Total SBQ points	4 ± 1.66 (range 1–8)

### Correlation analysis between ODI, SBQ, and AHI

There were significant positive correlations between the AHI, ODI, and SBQ mutually for patients with OSA overall. The ODI highly correlated with the AHI (*p* < 0.05 and *r* = 0.914) whilst the SBQ (*p* < 0.05, *r* = 0.357) showed a weaker positive correlation with AHI. It was also found that as the ODI score increases, so did the SBQ score (*p* < 0.05 and *r* = 0.451). This suggests that as AHI scores increase so does ODI and SBQ. However, the relationship is stronger between ODI and AHI. Table 2 displays correlation analyses between ODI and AHI for different OSA severities. The analyses found no significant correlations between the SBQ and AHI among the total sample. In contrast, AHI was positively correlated with the ODI for moderate and severe OSA patients (Spearman *ρ* of 0.493 and 0.796, respectively).

**Table 2:** Correlations between oxygen desaturation index and STOP-BANG questionnaire for apnea–hypopnea index ≥ 5, ≥ 15, or ≥ 30

Groups	Values	SBQ	AHI
AHI ≥ 5	AHI	0.136	-
	ODI	0.367*	0.297
AHI ≥ 15	AHI	− 0.047	-
	ODI	0.235	0.493*
AHI ≥ 30	AHI	0.235	-
	ODI	0.415	0.796**

^*^p < 0.05

### Evaluating the values of SBQ ≥ 3, ODI ≥ 5, and their combination

The diagnostic accuracy of ODI ≥ 5, SBQ ≥ 3, their combination, and a logistic regression model is presented in Table 3. Overall, SBQ ≥ 3 precisely screened 62% of OSA patients (AHI/REI ≥ 5/h). The sensitivity of SBQ increased as the severity of OSA increased (mild 90%, moderate 94%, and severe 100%). Similarly, the NPV increased (73%, 92%, and 100%, respectively). In contrast, the specificity of SBQ decreased as the severity of OSA increased (mild 31%, moderate 25%, severe 22%). Similarly, the PPV decreased (59%, 32%, and 14%, respectively). This indicates that the SBQ alone may be susceptible to accepting false positives as the severity of AHI increases.

**Table 3:** Diagnostic accuracy of SBQ ≥ 3 and ODI ≥ 5, the combination, and a logistic regression model to detect OSA

	Sensitivity	Specificity	PPV	NPV
AHI/REI ≥ 5/h (*n* = 70)				
SBQ ≥ 3	90%	31%	59%	73%
ODI ≥ 5	91%	80%	84%	89%
Combination of SBQ ≥ 3 with ODI ≥ 5	82%	85%	86%	81%
Logistic regression model (SBQ + ODI)	91%	60%	84%	89%
AHI/REI ≥ 15/h (*n* = 35)				
SBQ ≥ 3	94%	25%	32%	92%
ODI ≥ 5	94%	57%	44%	96%
Combination of SBQ ≥ 3 with ODI ≥ 5	88%	63%	47%	94%
Logistic regression model (SBQ + ODI)	89%	64%	47%	94%
AHI/REI ≥ 30/h (*n* = 15)				
SBQ ≥ 3	100%	22%	14%	100%
ODI ≥ 5	100%	47%	19%	100%
Combination of SBQ ≥ 3 with ODI ≥ 5	100%	55%	21%	100%
Logistic regression model (SBQ + ODI)	100%	55%	21%	100%

Abbreviations: *NPV*, negative predictive value; *PPV*, positive predictive value; sensitivity = TP / (TP + FN), specificity = TN / (TN + FP), PPV = TP / (TP + FP), NPV = TN / (TN + FN)

For ODI ≥ 5, the accuracy of the classification rate was 91% for OSA patients (AHI ≥ 5/h) which was much higher than SBQ (62%). The sensitivity increased as the severity of OSA increased (mild 91%, moderate 94%, severe 100%), and the NPV also increased (89%, 96%, and 100%, respectively). The specificity of ODI decreased as the severity of OSA increased (mild 80%, moderate 57%, severe 47%), and the PPV decreased (84%, 44%, and 19%, respectively).

The combination of SBQ with ODI and a logistic regression model (SBQ + ODI) showed the best performance of specificity in all severity levels of OSA (mild 85%, moderate 63%, severe 55%). There was also a decrease in PPV as the severity of AHI increased. However, these values were still higher than SBQ and ODI alone (mild 86%, moderate 47%, severe 21%). One consequence of this combination was that the sensitivity was slightly lower when ODI and SBQ measures were evaluated separately.

### The ROC of the SBQ ≥ 3 and ODI ≥ 5

**Fig. 2:**
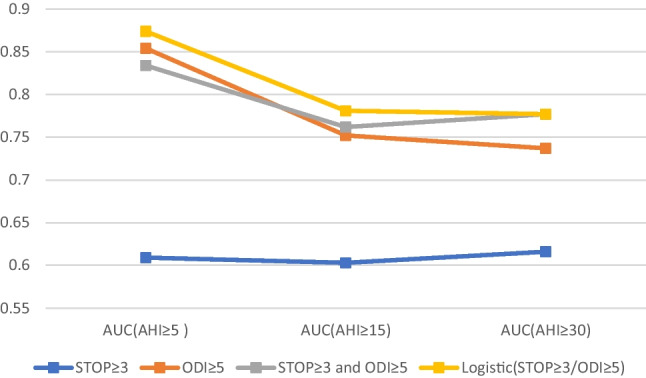
Area under curve (AUC) of the 4 scores at AHI/REI cut-off of 5, 15, and 30 event/h

**Fig. 3:**
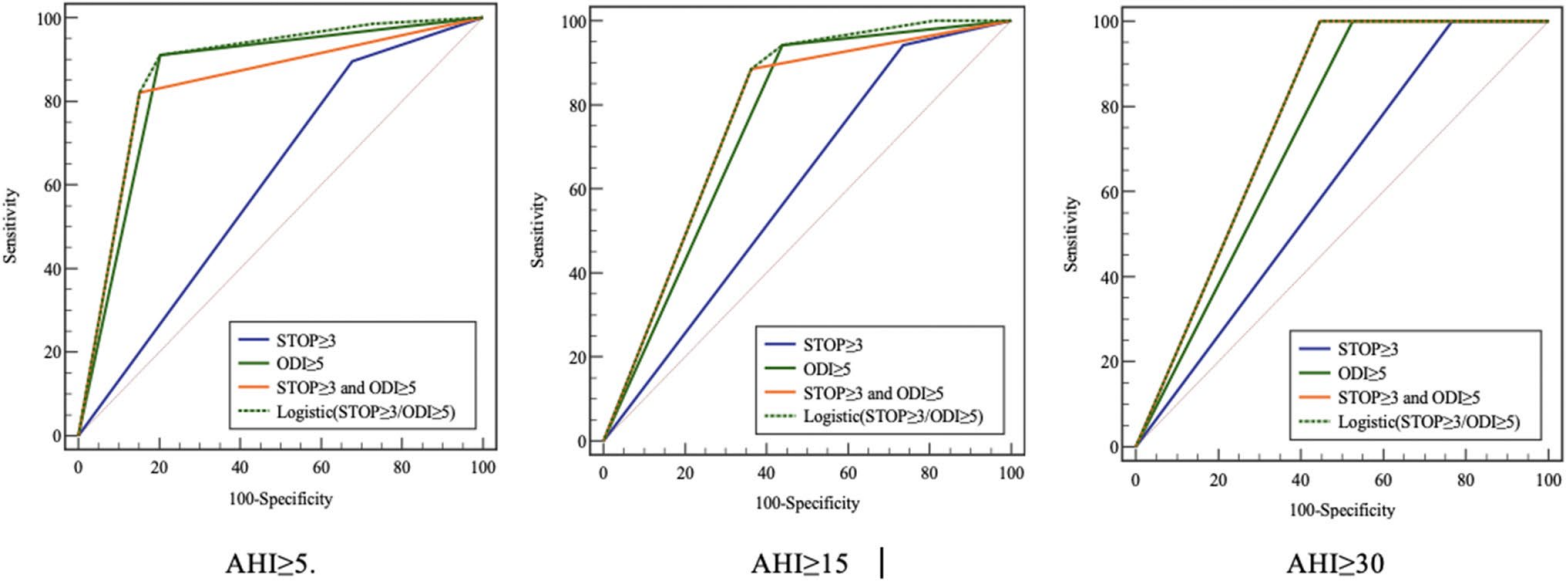
Receiver operator curve (ROC) analyses of a combination of STOP-Bang questionnaire with oxygen desaturation index versus STOP-Bang questionnaire and oxygen desaturation index alone in AHI ≥ 5, AHI ≥ 15, and AHI ≥ 30

See Figs. 2 and 3 for the ROC analyses set with ODI cut-off 5 and SBQ cut-off 3 across all severity levels of OSA. When the AHI cut-off was either 5, 15, or 30, the average AUC for ODI alone was 0.85, 0.75, and 0.74; for the combination of SBQ and ODI, it was 0.84, 0.76, and 0.77, and the logistic regression model showed 0.87, 0.78, and 0.77, respectively. According to the *Z*-test, all of which were significantly larger (*p* < 0.05) than SBQ ≥ 3 (0.61, 0.60, 0.62). However, when the SBQ was combined with ODI, it did not significantly contribute to the value of ODI (*p* ≥ 0.46).

In summary, ODI ≥ 5 had higher sensitivity, specificity, and a larger AUC than SBQ ≥ 3 for screening all OSA. Furthermore, ODI showed higher sensitivity, a slightly lower specificity, and the same overall value as the logistics model.

### The optimal cut-off of the SBQ and ODI

See Table 4 for the ROC analyses characteristics on ODI and SBQ across all severity levels of OSA. The optimal cut-off score for the SBQ was 5 for all severities of OSA. For ODI, the optimal cut-off scores were 8, 11, and 22, indicating a maximal AUC of 0.929, 0.906, and 0.988 for mild, moderate, and severe OSA, respectively. Both the individual ODI and the combination of ODI with SBQ analyses showed statistically significant diagnostic values for OSA across all severity levels. Also, they both had a larger and approximately similar ROC than the individual SBQ analysis. For the identification of mild OSA, SBQ AUC = 0.688 [0.596–0.779], ODI AUC = 0.929 [0.714–0.940], and both combined AUC = 0.929 [0.714–0.940] with a Youden index of 0.813. For the identification of moderate OSA, SBQ AUC = 0.685 [0.58–0.79], ODI AUC = 0.906 [0.838–0.975], and both combined AUC = 0.907 [0.839–0.975] with a Youden index of 0.705. For the identification of severe OSA, SBQ AUC = 0.736 [0.598–0.874], ODI AUC = 0.988 [0.973–1], and both combined AUC = 0.988 [0.973–1] with a high Youden index of 0.946. Overall, it is shown here that the output from the combination of SBQ and ODI analysis is relatively similar to the individual ODI output with a slight difference in both the AUC and Youden index. ODI at the optimal cut-off value could display an almost perfect diagnostic ability for all severities of OSA.

**Table 4:** Receiver operator curve (ROC) analyses of oxygen desaturation index and STOP-Bang questionnaire for AHI ≥ 5, ≥ 15, ≥ 30

AHI	Variable	AUC	SE	95% Confidence interval		Cut-off	Sensitivity	Specialty	Youden index
				Low area	Upper area				
≥ 5	SBQ	0.688	0.047	0.596	0.779	4.500	0.493	0.780	0.272
	ODI	0.929	0.025	0.881	0.978	7.600	0.896	0.932	0.828
	SBQ with ODI	0.929	0.025	0.881	0.978	0.506	0.881	0.932	0.813
≥ 15	SBQ	0.685	0.053	0.580	0.790	4.500	0.543	0.703	0.246
	ODI	0.906	0.035	0.838	0.975	10.850	0.914	0.802	0.716
	SBQ with ODI	0.907	0.035	0.839	0.975	0.165	0.914	0.791	0.705
≥ 30	SBQ	0.736	0.070	0.598	0.874	4.500	0.714	0.679	0.393
	ODI	0.988	0.008	0.973	1.000	22.250	1.000	0.929	0.929
	SBQ with ODI	0.988	0.008	0.973	1.000	0.078	1.000	0.946	0.946

## Discussion

This study showed that using the ODI alone may serve as a good preliminary assessment of OSA whilst waiting for a full diagnosis from HSAT or PSG. However, the combination of SBQ failed to increase the diagnostic value of ODI, and the rate of accepting a false negative slightly increased.

A positive correlation was detected for both ODI and SBQ with AHI. This suggests that as the AHI severity increases, the SBQ and the ODI scores decrease. When breaking this down by severity of AHI, a strong positive relationship can be seen in ODI alone with moderate and severe OSA whilst the SBQ displayed non-significant relationships. On the other hand, SBQ and ODI had no significant positive correlation when diagnosing mild OSA. This suggests that ODI alone would be better at diagnosing moderate and severe OSA than SBQ. Considering possible moderating factors, the low correlation between SBQ and AHI might have been affected by the small sample. Another reason could be that there was an uneven gender split of 74% of males compared to a low proportion of females (26%).

Our findings indicated that SBQ alone and ODI alone demonstrated high sensitivity for each severity of OSA classified by the AHI. However, the specificity scores of SBQ for each severity level remained low. In terms of the PPV and NPV, there is a greater likelihood of accepting false positives than false negatives which means that if SBQ alone were used individually with AHI, then there would be a greater possibility of misdiagnosing the severity of OSA, especially moderate and severe as their specificity scores are low. Nagappa et al. [21] conducted a meta-analysis to measure the validity of the SBQ as a screening tool for severe OSA and found high validity. They also reported that when the severity of AHI increased, the sensitivity of the SBQ increased whilst the specificity decreased, which is in line with the results shown in this current study. Alternatively, when ODI alone or both diagnostic tools were combined and compared against the AHI, it showed an increase in specificity across all severities whilst the sensitivity for each severity decreased slightly. This suggests that ODI or the combination could be beneficial to diagnosing OSA in terms of increasing specificity. Consequently, ODI displayed a better value and should be the preferred way to initially screen OSA patients, whereas SBQ should be considered in large populations as it is inexpensive and less time-consuming than oximetry monitoring.

Previous studies have focused on improving the PPV and specificity of the SBQ by combining it with different tools [22, 23]. Senaratna et al. [24] found that when combining the SBQ with the Epworth Sleepiness Scale ≥ 8, the specificity was high (94–96%) and the sensitivity was low (36–51%). In 2012, Chung et al. [25] demonstrated that the serum HCO3-levels only increased the specificity of the SBQ in predicting moderate/severe OSA. Dette et al. [26] combined SBQ scores with Mallampati scores, which failed to improve the specificity in predicting SBD. Another study examined the predictive ability of OSA screening questionnaires versus oximetry for CPAP therapy initiation and identified that oximetry performed better than questionnaires (Berlin questionnaire, Epworth Sleepiness Scale, and SBQ) in predictive ability. However, their combination did not improve their predictive value [27].

As mentioned previously, a strong correlation was found between ODI and AHI in the multiple logistic analysis (*r* = 0.914), which suggests that this model showed a good performance to screen all severities of OSA. Therefore, overnight oximetry seems to be an inexpensive, readily available, and straightforward tool to screen for OSA. Previous studies have also emphasized the accuracy of ODI as a tool to detect SBD and suggested it should be an essential assessment criterion for diagnosis [9, 27, 28]. Mashaqi et al. [29] found that the use of nocturnal oximetry measures (ODIPOx) improved the accuracy of SBQ in severe OSA in both inpatient and outpatient settings. Our study also showed this; however, the cost of improving the diagnostic ability of SBQ reduced the diagnostic ability of the ODI. Therefore, combining SBQ with ODI is suboptimal compared to using ODI alone to diagnose OSA.

The diagnostic ability of SBQ, ODI, and their combination at different cut-offs has been investigated previously. Chung et al. [30] found that ODI from a high-resolution nocturnal oximeter was a sensitive and specific tool to detect undiagnosed SBD in surgical patients. Their cut-off values for ODI to predict AHI > 5, AHI > 15, and AHI > 30 were ODI > 5, > 15, and > 30. This current study showed that an ODI cut-off of 8, 11, and 22 had a maximal AUC for mild, moderate, and severe OSA. The cut-off scores for SBQ were also adjusted to maximize the diagnostic ability of all assessments. When both ODI alone and the combination of ODI and SBQ were maximized, they both had an excellent discriminative ability to predict mild, moderate, and severe OSA in all patients. ODI combined with SBQ displayed the most significant AUC and highest Youden index in patients with severe OSA. This suggests that when ODI cut-off scores are optimized, ODI could evaluate all degrees of severity of OSA accurately. Therefore, these optimized cut-off scores may need to be considered if this combination was to be used to maximize the perfomance of ODI in OSA.

### Limitations

A limitation of this study is the small sample size. Some of the patients may have given more extreme answers on the SBQ than others. This could skew the accuracy and comparability of the value. Therefore, larger studies are needed to confirm the findings of this study.

In addition, the correlation and ROC analyses in this study reported contrasting results for the relationship between the AHI with the combination of ODI and SBQ. However, this was mostly dependent on different cut-off values of the SBQ calculated for different severities of OSA.

A further limitation is that this study evaluated the ODI adopted from sleep study reports instead of portable nocturnal pulse oximetry. This means that the ODI was not scored independently from the AHI/REI, whereas it is possible to do this with portable oximetry devices. These devices can record more data and provide detailed information about the oxygen level at night (including a detailed graph and 4% desaturation events). Therefore, the value produced by these new devices should be studied further.

## Conclusion

This study attempted to improve the diagnostic ability of the SBQ in screening and predicting the degree of OSA by combining it with ODI. According to the findings, ODI alone had a higher sensitivity and specificity than SBQ, and the combination with SBQ failed to provide additional diagnostic value. Therefore, a simple portable nighttime pulse oxygen saturation monitoring device may be the preferred way to initially screen for potential OSA with comparatively low cost compared to PSG.

## Data Availability

All data generated or analyzed during this study are included in this published article.
